# Laparoscopic Localization and Enucleation of Multiple Insulinomas Using a Combination of Indocyanine Green Fluorescence Imaging and Intraoperative Ultrasound

**DOI:** 10.1245/s10434-026-19454-y

**Published:** 2026-04-30

**Authors:** Jia Li, Shihao Jiang, Lei Zhou, Yuansong He, Chao Chen, Songling Liu, Jun Yang, Minjie Zhou, Yi Liu, Wei Cheng

**Affiliations:** 1 Department of Hepatobiliary Surgery, Hunan Provincial People’s Hospital, The First Affiliated Hospital of Hunan Normal University, Hunan, China; 2 The First Clinical Medical College, Guangdong Medical University, Zhanjiang, China

## Abstract

**Introduction:**

Enucleating multiple insulinomas adjacent to the main pancreatic duct poses significant risks of parenchymal injury and postoperative complications. While indocyanine green (ICG) fluorescence aids in tumor localization, excessive background fluorescence and diminished intensity during prolonged procedures can obscure tumor margins.

**Methods:**

A 38-year-old male presented with multiple insulinomas located in the pancreatic head, tail, and uncinate process. The head and tail lesions were within 1 mm of the main pancreatic duct. Laparoscopic enucleation was planned using combined ICG fluorescence and intraoperative ultrasound (IOUS). The patient received 25 mg of intravenous ICG 24 hours preoperatively.

**Results:**

Intraoperative probing revealed no fluorescence from the preoperative ICG dose. Subsequently, a 12.5 mg intravenous ICG bolus was administered. Tumor enhancement was achieved in 15 seconds, and distinct tumor demarcation appeared at 30 minutes as background fluorescence dissipated. Guided by real-time ICG and IOUS, the tumors were safely enucleated using cold sharp dissection near the duct. Intraoperative portal vein blood sampling confirmed a significant decline in insulin and C-peptide levels. Blood loss was 50 mL, and operative time was 200 minutes. The patient was discharged on postoperative day 4 without pancreatic fistula.

**Conclusion:**

Integrating an intraoperative 12.5 mg ICG bolus with IOUS provides precise localization and enables safe, parenchyma-sparing enucleation of multiple insulinomas near the main pancreatic duct. Preoperative ICG administration 24 hours prior is not recommended.

**Supplementary Information:**

The online version contains supplementary material available at 10.1245/s10434-026-19454-y.

Establishing a precise anatomical enucleation plane constitutes one of the foremost challenges in laparoscopic insulinoma enucleation.^1,2^ For insulinomas intimately adherent to the main pancreatic duct, discernible boundaries are virtually absent, rendering the enucleation process highly susceptible to inadvertent tissue injury and thereby elevating the risk of postoperative complications.^3^ Furthermore, multiple insulinomas necessitate precise enucleation of multiple lesions while minimizing parenchymal injury, thereby resulting in longer operative durations, heightened procedural complexity, elevated surgical risks, and greater technical demands on the operating surgeon compared with solitary insulinomas.^4,5^ Intraoperative indocyanine green (ICG) fluorescence imaging effectively delineates the boundaries between tumor and normal tissue, thereby providing valuable adjunctive support for laparoscopic insulinoma enucleation and localization.^6,7^ However, this modality is often confounded by excessive background fluorescence, and the protracted operative duration in cases of multiple insulinomas may lead to diminished fluorescence intensity in the later stages, both of which can impede clear visualization of tumor-normal tissue interfaces.^8,9^ Therefore, for multiple insulinomas intimately adherent to the main pancreatic duct (particularly those with tumors located less than 1 mm from the duct), ICG fluorescence imaging combined with intraoperative ultrasound (IOUS) is particularly essential for tumor localization. Video 1 demonstrates a practical workflow integrating ICG fluorescence imaging with IOUS to facilitate localization and parenchyma-sparing enucleation of multiple insulinomas, particularly for lesions adjacent to the main pancreatic duct.

A 38-year-old male patient was admitted to our institution with a diagnosis of multiple insulinomas. Given a history of parathyroid adenoma, multiple endocrine neoplasia type 1 (MEN1) was clinically suspected, although genetic testing was declined after informed consent. Preoperative computed tomography (CT) and magnetic resonance cholangiopancreatography (MRCP) revealed multiple insulinomas located in the uncinate process of the pancreas, the pancreatic head, and the pancreatic tail. The tumors in the pancreatic head and tail were situated less than 1 mm from the main pancreatic duct, while the uncinate process lesion was 3 mm from the duct. To achieve complete tumor excision while preserving the integrity of the main pancreatic duct, we planned a laparoscopic enucleation of multiple insulinomas under the guidance of ICG fluorescence imaging combined with IOUS. The study was conducted in accordance with the Declaration of Helsinki and its subsequent amendments. The study protocol was approved by the Ethics Committee of Hunan Provincial People’s Hospital (The First Affiliated Hospital of Hunan Normal University) (number: 2024-133). Written informed consent was obtained from the patient for publication of this article, accompanying images and video. A copy of the written consent is available for review by the editorial office of this journal. The three-dimensional (3D)/4K ICG fluorescence imaging was performed intraoperatively with the Rubina imaging system (Storz, Tuttlingen, Germany).

ICG at a dose of 25 mg was administered intravenously 24 h prior to surgery. Upon exposure of the first tumor intraoperatively, fluorescence probing revealed no enhancement in either the tumor or the surrounding normal pancreatic tissue. A subsequent intravenous bolus of ICG 12.5 mg was then administered. Fluorescence initially manifested in the vasculature and normal pancreatic parenchyma, subsequently accumulating within the tumor tissue. Approximately 15 s later, the tumor achieved complete enhancement; at 1 min, tumor fluorescence intensity surpassed that of the normal pancreas; and at 30 min, fluorescence in the normal pancreatic tissue had dissipated, leaving clear tumor demarcation. Under real-time ICG fluorescence imaging integrated with IOUS (Fig. 1), precise tumor localization and enucleation along tumor margins were facilitated. To minimize lateral thermal spread, the ultrasonic shears were applied with the active blade oriented away from the main pancreatic duct. For the critical tumor-duct interface (final 1–2 mm), energy devices were suspended. Instead, cold sharp dissection and blunt spreading were used exclusively to safely separate the tumor. Following the enucleation of all tumors, portal vein (PV) blood was sampled intraoperatively for real-time measurement of serum insulin and C-peptide levels, which demonstrated a significant decline compared with preoperative values. Operative duration was 200 min, with an estimated blood loss of 50 mL. Pathological examination of the three resected specimens confirmed insulinomas. Immunohistochemistry showed all three lesions were positive for insulin, and diffuse membranous Glut2 expression was observed in all tumors, verifying their functional nature (Fig. 2). Drain fluid amylase levels were transiently elevated on postoperative day 1 (752 U/L) but normalized by day 3 (88 U/L). No clinically relevant postoperative pancreatic fistula occurred according to ISGPS criteria. The patient was discharged on postoperative day 4 without any complications.

**Fig. 1:**
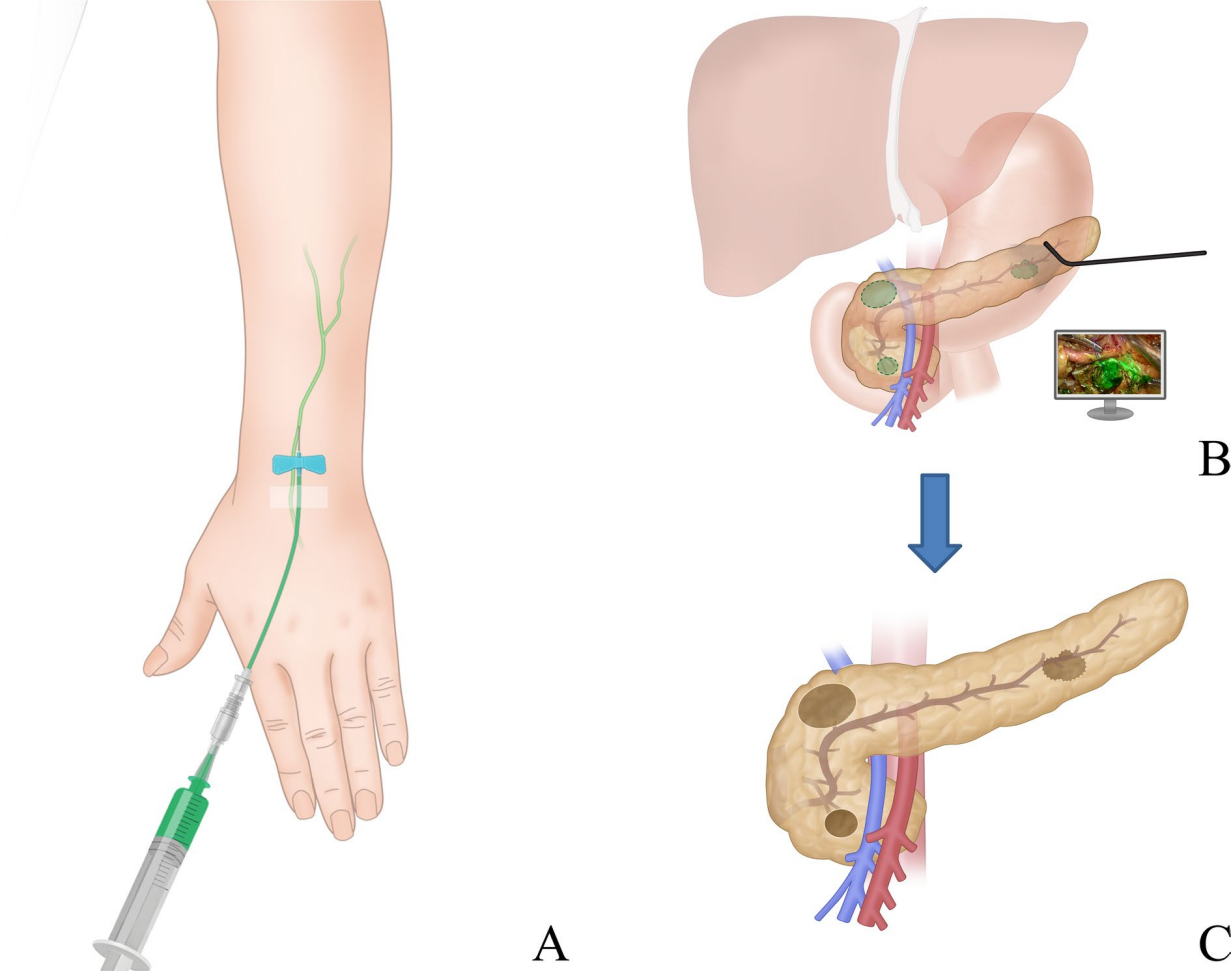
Workflow for laparoscopic enucleation of multiple insulinomas guided by the combination of indocyanine green (ICG) fluorescence imaging and intraoperative ultrasound (IOUS). **a** Diagram showing the ICG administration timeline. Since the preoperative dose (25 mg) failed to visualize the tumors, a subsequent 12.5 mg intraoperative bolus was administered. **b** Subsequent ICG fluorescence imaging, in combination with IOUS, guided the precise enucleation procedure. **c** Final view after successful enucleation of three insulinomas under the dual guidance of ICG fluorescence and IOUS

**Fig. 2:**
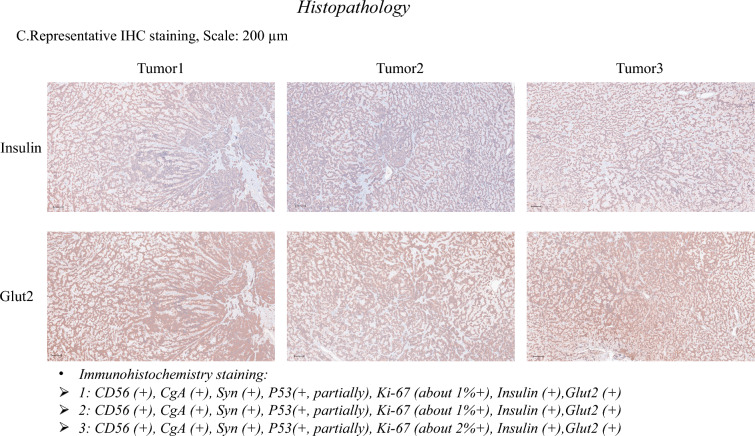
Immunohistochemical staining demonstrating positive Insulin and Glut2 expression in the resected insulinomas

In summary, this study demonstrates that for the parenchyma-sparing enucleation of benign pancreatic tumors, such as multiple insulinomas, the integration of ICG fluorescence imaging with ultrasound guidance is recommended. This multimodal approach facilitates accurate tumor localization and demarcation of tumor-normal tissue interfaces, thereby mitigating the risk of incomplete resection or excessive parenchymal loss (especially in cases involving small or multifocal lesions) while preserving the integrity of the main pancreatic duct. Such a strategy is consistent with established evidence-based criteria for local excision, eliminating the requirement for pancreatic duct reconstruction. Furthermore, preoperative ICG administration 24 h prior to surgery is not recommended, whereas intraoperative intravenous injection of 12.5 mg ICG effectively visualizes insulinomas. Finally, to assess the completeness of multiple insulinoma enucleation, beyond reliance on preoperative imaging, intraoperative fluorescence, and ultrasound, post-enucleation sampling of portal venous blood for real-time measurement of serum insulin and C-peptide levels, with confirmation of a significant decline, serves as an effective adjunctive metric to verify comprehensive tumor resection.

## Supplementary Information

Below is the link to the electronic supplementary material.

Supplementary file 1 (MP4 97163 KB)
